# Interactions of genetic risks for autism and the broad autism phenotypes

**DOI:** 10.3389/fpsyt.2023.1110080

**Published:** 2023-03-21

**Authors:** Lijie Dong, Yijing Wang, Xiaomeng Wang, Tengfei Luo, Qiao Zhou, Guihu Zhao, Bin Li, Lu Xia, Kun Xia, Jinchen Li

**Affiliations:** ^1^Bioinformatics Center and National Clinical Research Centre for Geriatric Disorders, Department of Geriatrics, Xiangya Hospital, Central South University, Changsha, Hunan, China; ^2^Centre for Medical Genetics and Hunan Key Laboratory of Medical Genetics, School of Life Sciences, Central South University, Changsha, Hunan, China; ^3^Department of Neurology, Xiangya Hospital, Central South University, Changsha, Hunan, China

**Keywords:** autism spectrum disorder, polygenic risk score, *de novo* variants, broad autism phenotypes, sex, liability

## Abstract

**Background:**

Common polygenic risk and *de novo* variants (DNVs) capture a small proportion of autism spectrum disorder (ASD) liability, and ASD phenotypic heterogeneity remains difficult to explain. Integrating multiple genetic factors contribute to clarifying the risk and clinical presentation of ASD.

**Methods:**

In our study, we investigated the individual and combined effects of polygenic risk, damaging DNVs (including those in ASD risk genes), and sex among 2,591 ASD simplex families in the Simons Simplex Collection. We also explored the interactions among these factors, along with the broad autism phenotypes of ASD probands and their unaffected siblings. Finally, we combined the effects of polygenic risk, damaging DNVs in ASD risk genes, and sex to explain the total liability of ASD phenotypic spectrum.

**Results:**

Our findings revealed that both polygenic risk and damaging DNVs contribute to an increased risk for ASD, with females exhibiting higher genetic burdens than males. ASD probands that carry damaging DNVs in ASD risk genes showed reduced polygenic risk. The effects of polygenic risk and damaging DNVs on autism broad phenotypes were inconsistent; probands with higher polygenic risk exhibited improvement in some behaviors, such as adaptive/cognitive behaviors, while those with damaging DNVs exhibited more severe phenotypes. Siblings with higher polygenic risk and damaging DNVs tended to have higher scores on broader autism phenotypes. Females exhibited more severe cognitive and behavioral problems compared to males among both ASD probands and siblings. The combination of polygenic risk, damaging DNVs in ASD risk genes, and sex explained 1–4% of the total liability of adaptive/cognitive behavior measurements.

**Conclusion:**

Our study revealed that the risk for ASD and the autism broad phenotypes likely arises from a combination of common polygenic risk, damaging DNVs (including those in ASD risk genes), and sex.

## Introduction

Autism spectrum disorder (ASD) is a neurodevelopmental condition with high heritability. Both *de novo* variants (DNVs) and polygenic risk have been implicated in the development of ASD, but their burden may differ. DNVs have been identified in a subset of individuals with ASD, particularly in those with intellectual disability and/or severe language impairments (1). On the other hand, polygenic risks are thought to be more common in individuals with ASD, particularly those without intellectual disability. While the burden of polygenic risk factors may be less severe than rare genetic mutations, they may still have a significant impact on affected individuals, as well as on their families and communities (2, 3). Although common polygenic risk was estimated to explain up to 52% of ASD heritability and DNVs in genes have a large effect size, the combined contribution of polygenic risk and all DNVs is relatively modest, explaining only around 5% of ASD variance (2, 4). There is a sex-based difference in the incidence rate of ASAD, with males being four times more likely than females to be diagnosed. However, this ratio has been decreasing in recent years, possibly due to increased clinical attention to ASD in females (5). The “female protective effect” under a liability threshold model in ASD may also contribute to the sex difference, as females may require more genetic risks than males to meet the ASD diagnostic threshold (6). Females with ASD had a significantly increased rare variant risk score and common variant risk score than males with ASD (4). What’s more, families with a history of ASD have a greater polygenic risk for ASD than the general population and siblings of females diagnosed with ASD have a higher likelihood of developing ASD compared to siblings of males diagnosed with ASD (3).

ASD is characterized by impaired social interaction and communication, repetitive behavior, and restricted interests. Besides these core symptoms of ASD, patients may also present with neuropsychiatric comorbidities, such as intellectual disability and developmental delays (7, 8). The broader autism phenotype describes a range of traits that resemble ASD, but are considered subclinical or not enough to qualify for a diagnosis of ASD (9). These traits suggest a genetic liability for ASD-related traits in families, and it is estimated that at least 10–20% of siblings of children diagnosed with ASD exhibit such characteristics (10). A recent study showed the effects of most genetic factors on behavioral traits were similar in females and males (4).

A polygenic risk score (PRS) is a calculation of an individual’s genetic susceptibility to a specific trait or disease based on their genotype profile and relevant genome-wide association study (GWAS) data (11). PRS have demonstrated a modest ability to distinguish between ASD case–control groups and provide continuous analysis of complex phenotypes (2, 12). Studies have found positive correlations between PRS for ASD and intelligence quotient (IQ) and educational attainment, with higher PRS associated with a lower likelihood of co-occurring developmental disabilities in ASD individuals (13, 14). In contrast, subjects carrying damaging DNVs have lower non-verbal IQs than those without damaging DNVs (15). Moreover, an increasing number of damaging DNVs is associated with a higher risk of adverse neurodevelopmental outcomes (16). The liability-threshold model indicates that a total genetic load can be reached by combinations of different variants to meet the diagnostic criteria (4). Investigating the effects of PRS, DNVs, and sex on ASD risk and phenotype in probands and their siblings can aid in understanding the heterogeneity of ASD.

In this study, we analyzed data from 2,591 ASD simplex families to examine the effects of common polygenic risk, DNVs, damaging DNVs, and those occurring in ASD risk genes. We calculated individual PRS and explored the individual and combined effects of PRS, damaging DNVs, and sex on ASD and the broad autism phenotype in siblings. Additionally, we estimated the impact of PRS, damaging DNVs occurring in ASD risk genes, and sex on core ASD phenotypes separately and in combination to predict ASD liability.

## Materials and methods

### Samples and quality control

Participants were drawn from the Simons Simplex Collection (SSC) in biological family groups, each family had one child with ASD, and unaffected parents and siblings (17). Samples were genotyped on one of three Illumina platforms: 1Mv1, 1Mv3, or Omni2.5 (Illumina, San Diego, CA) and included 10,220 individuals from 2,591 families. Elise Robinson used the Ricopili pipeline to perform imputation, which is publicly available and has been reported on extensively (16, 18). Then, we excluded SNPs that were poorly imputed (info score ≤ 0.7). Combined with all arrays after imputation, 10,206 individuals (2,601 ASD probands) and 5,356,600 SNPs were included. Next, we adopted rigorous quality control using PLINK software version 1.9 (19). SNPs with missingness rate ≥ 0.02, minor allele frequency ≤ 0.01, and unsatisfied Hardy–Weinberg equilibrium test (*p* < 1e-6) were filtered out. Individuals with a missingness rate ≥ 0.02 and heterozygosity rate deviating more than 3 standard errors from the means were removed. Finally, 9,797 samples (2,494 ASD probands, 2,341 unaffected siblings), and 3,650,393 SNPs were analyzed. We performed a transmission disequilibrium test and calculated the inflation factor based on *p*-values. The lambda (1.06) was close to 1, indicating immunity to confounding factors, therefore, no further adjustment for the array platform was made (Supplementary Figure S1). Whole exome sequencing data was also obtained from the same cohort, which included sequences for 2,508 affected children and 1,911 unaffected siblings. In the analysis of the interactions between polygenic risk and DNVs, 2,250 ASD probands and 1,740 unaffected siblings had overlapping genotype arrays and whole exome sequencing data.

### Polygenic risk scoring

We utilized PRS-CS (20) to calculate PRS, which provides a quantitative measure of individual genome-wide common variant predisposition (or ‘risk’) for a trait or disease (20). This method estimates posterior SNP effect sizes under continuous shrinkage priors using GWAS summary statistics and an external linkage disequilibrium reference panel. We obtained summary statistics from a recent ASD GWAS involving over 18,000 cases and almost 28,000 controls (2) and used the European sample from the 1,000 Genomes Project phase 3 as the linkage disequilibrium reference panel to estimate the posterior effect size for each SNP (21). PLINK version 1.9 was used to sum all SNPs to individual-level polygenic scores and shift all scores to mean zero for a convenient comparison of different groups. PRSs were generated for 9,797 individuals with 3,659,780 SNPs. To reduce case–control sample size bias, we only considered ASD probands and unaffected siblings when comparing the PRS difference. The PRS distribution plot and receiver operating characteristic curve plot in R were drawn using RStudio (Integrated Development for R; RStudio, PBC, Boston, MA). The receiver operating characteristic curve represents the diagnostic ability of a binary classifier system as its discrimination threshold changes.

### DNV collection and annotation

DNVs, including *de novo* single nucleotide variants from Iossifov et al. (15) and *de novo* copy number variants from Sanders et al. (22), were analyzed for the SSC cohort of 2,508 affected children and 1911 unaffected siblings. Gene4denovo is an integrated database and analysis platform used to identify DNVs, perform custom annotations, and prioritize pathogenetic variants and risk genes (23). REVE is a computational method developed to predict the pathogenicity of missense variants, showing the best overall performances with all the benchmark data (24). We defined damaging missense variants as REVE ≥0.7, and tolerant missense variants as REVE <0.7. Loss-of-function variants were defined as frameshifting, stop gain/loss, and splicing variants. The above *de novo* variants were annotated using Gene4Denovo. Damaging missense variants, Loss-of-Fuction variants, and copy number variants consolidated into damaging DNVs, which represent the modest to high effect size. We selected 227 high-confidence ASD risk genes from SFARI with a score of 1 (2021-09) (25) and the SPARK gene list reported in recent literatures (2020-07) (26) (Supplementary Table S1). We mapped damaging DNVs occurred in ASD risk genes, which were considered more deleterious for ASD.

### Broad autism phenotypes

We first focused on the core clinical phenotypes of ASD according to DSM-5 diagnosis criteria, which consist of impaired social communication and restricted, repetitive, and/or sensory behaviors or interests (27). The ASD core phenotypes were divided into four categories in the SSC version 15.3 phenotype dataset, namely adaptive/cognitive behaviors, language/communication social behaviors, repetitive and restricted behaviors, and problem behaviors. Each category contained multiple measurements from various instruments, and all measurements of a continuous distribution represented the total score of all items of the instruments to reflect phenotype performance. Higher scores in adaptive/cognitive behaviors indicated better performance, while higher scores in other core phenotypes indicated more severe symptoms (Supplementary Table S2).

In addition to the core phenotypes, we analyzed ASD broad phenotypes recorded in the SSC database. Autism Diagnostic Observation Schedule (ADOS) assesses ASD symptom severity across the domains of social reciprocity, language use, and restricted and repetitive behaviors and interests for diagnosing (28); Autism Diagnostic Interview-Revised (ADI-R), a parent interview that assesses the child’s symptoms related to ASD (29); Child Behavior Checklist (CBCL), a parent-report questionnaire used to assess emotional and behavioral problems in children (30); Comprehensive Test of Phonological Processing (CTOPP) to assesses linguistic competence; Teacher Report Form (TRF), OR Caregiver-Teacher Report Form (CTRF) (31) assesses problem behavior, academic performance, and adaptive functioning (32); Differential Ability Scales-II (DAS-II) provides a psychologist with insight into how a child processes information (33); Developmental Coordination Disorder Questionnaire (DCDQ) designed to screen for coordination disorders in children, aged 5 to 15 years (34). Repetitive Behavior Scale-Revised (RBS-R) to measure restricted and repetitive behaviors (35); Evaluate communication skills and social functioning in children by Social Communication Questionnaire (SCQ) and Social Responsiveness Scale (SRS) (36, 37) Vineland Adaptive Behavior Scales (VABS-II) measure adaptive behavior and support the diagnosis of intellectual and developmental disabilities, autism, and developmental delays by parents (38) (Supplementary Table S3). We also analyzed the broad autism phenotypes in siblings using several instruments, including the Vineland Adaptive Behavior Scales (VABS-II), Child Behavior Checklist (CBCL), the Teacher Report Form (TRF), and the Caregiver-Teacher Report Form (CTRF), the Social Communication Questionnaire (SCQ), and the Social Responsiveness Scale (SRS). Our analysis aimed to compare potential sex differences and explore their relationship with PRS and DNVs (Supplementary Table S4).

### Statistical analysis

We conducted a series of regression analyses by using Rstudio and R packages. Simplex linear regression was used to model the relationship between PRS and the broad autism phenotypes of ASD and their siblings. Logistic regression was used to model (1) the interaction between PRS, DNVs, and damaging DNVs in ASD risk genes, and (2) the relationship between the level of DNV risk, with and without damaging DNVs in ASD risk genes, and ASD core phenotypes. We classified ASD probands with damaging *de novo* variants (DNVs) into three risk levels based on odds ratios: (1) low risk (score = 0, *n* = 1,516), (2) intermediate risk (score >0 and <3, *n* = 861), and (3) high risk (score ≥3, *n* = 131). We used the R package MASS (39) for ordered classification logistic regression to model the relationship between damaging DNVs risk and core ASD phenotypes, and the Brant test from the Brant package^1^ to test the assumption of proportional odds. We conducted a *t*-test to compare PRS between groups as the distribution of PRS passed the normality test. As for phenotype assessment scores, we used the rank sum test due to their skewness. We included sex, PRS, and damaging DNVs in ASD risk genes as covariates in the multiple linear regression analysis to predict the liability of ASD adaptive/cognitive phenotype. To account for multiple comparisons, we used the honestly significant difference method to adjust the *p*-value. The R^2^ statistic and adjusted Nagelkerke *R*^2^ were used to determine the proportion of variance in the liability scale and the overall proportion of variance in ASD adaptive/cognitive phenotype explained by sex, PRS, and damaging DNVs in ASD risk genes.

## Results

### Identifying the contributions of polygenic risk to ASD

**Figure 1:**
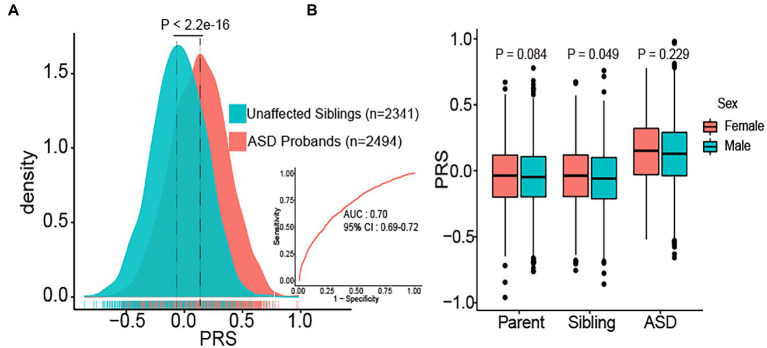
Distribution of polygenic risk score (PRS) in different subgroups. **(A)** The distribution of PRS in ASD probands (*n* = 2,494) and unaffected sibling controls (*n* = 2,341). PRS values were z-score scaled, and significance was determined using t-tests, receiver operating characteristic (ROC) curve of PRS results was presented. AUC: the area under the curve. **(B)** Boxplots of PRS burden in parents (*n* = 4,962), siblings (*n* = 2,341), and ASD probands (*n* = 2,494) are shown separately for females and males.

ASD probands (*n* = 2,494) had a significantly higher PRS burden than their unaffected siblings (*n* = 2,341) (*p* < 2.2e-16). The area under the curve value of the PRS model was 0.70 (95% CI = 0.69–0.72) (Figure 1A). We also compared PRS differences in sex by dividing all samples into three subgroups (parents, siblings, ASD probands). In all subgroups, females had a slightly higher PRS burden than males. In siblings, females had a significantly higher PRS burden (mean ± SD: −0.04 ± 0.23) than males (mean ± SD: −0.059 ± 0.23) (*p* = 0.048). Although sex in ASD probands lacks a significant difference, PRS in females (mean ± SD: 0.145 ± 0.135) was slightly higher than in males (mean ± SD: 0.127 ± 0.005) (*p* = 0.23) (Figure 1B). Moreover, we did not observe a significant difference in PRS between the siblings of females diagnosed with ASD and siblings of males diagnosed with ASD (Supplementary Figure S2). The statistical significance may have been impacted by the relatively small sample size of females with ASD (*n* = 332).

**Figure 2:**
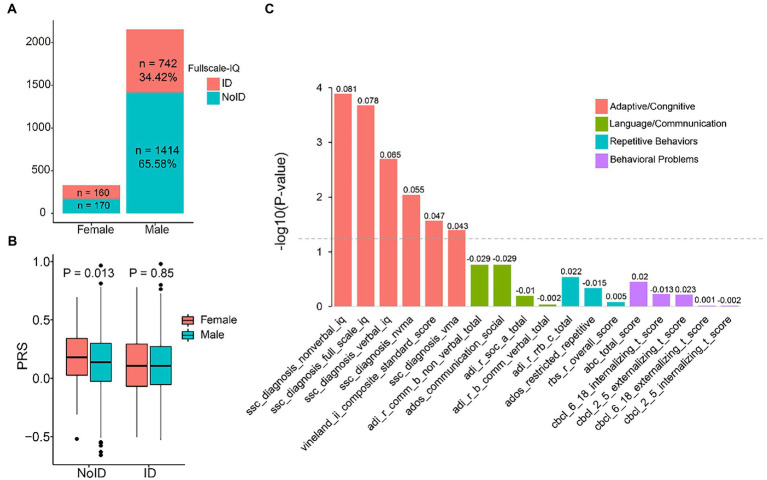
Relationship between polygenic risk score (PRS) and autism spectrum disorders (ASD) core phenotypes. **(A)** The proportion of probands with and without intellectual disability (ID) in females and females. **(B)** Boxplots of PRS in female and male probands with and without ID, group differences were tested *via* a *t*-test. **(C)** Correlations between PRS and ASD core phenotypes. The gray dotted line indicates a significance threshold of *P* ≤ 0.05. Correlation coefficients are displayed above the bars.

Next, we analyzed the association between PRS and ASD phenotype severity. We used full-scale IQ >75 to define intellectual disability in ASD probands. There were more males without intellectual disability (1.91:1), while there was almost no difference among females (1.06:1) (Figure 2A). We observed that in subjects without intellectual disability, the PRS of females was significantly higher than that of males (*p* = 0.013), but the difference disappeared in subjects with intellectual disability (Figure 2B). Similarly, cognitive impairment (Vineland score < 70) showed a similar tendency, with more males showing cognitive impairment features (1.53:1), and female PRS was higher than male PRS in terms of cognitive impairment (*p =* 0.02) (Supplementary Figure S3).

We then used linear regression to investigate the association between PRS and ASD core phenotypes. Our results showed that adaptive/cognitive behaviors were positively associated with PRS. Specifically, nonverbal IQ had a correlation coefficient of 0.081 (*p* = 0.0013), full-scale IQ had a correlation coefficient of 0.078 (*p* = 0.00021), and verbal IQ had a correlation coefficient of 0.065 (*p* = 0.002). Other adaptive and cognitive scores showed weaker associations. The correlation coefficient of Vineland’s adaptive behavior was 0.047 (*p* = 0.027), nonverbal mental age was 0.055 (*p* = 0.0091), and verbal mental age was 0.043 (*p* = 0.04). However, We did not observe any significant correlation between PRS and other ASD core phenotypes, such as language/communication, repetitive behaviors, and behavioral problems (Figure 2C).

We observed that the female phenotypes were generally more severe than the male phenotypes in each adaptive/cognitive behavior item (Supplementary Figure S4). This suggests that common polygenic risk may not be sufficient to explain the complexity of ASD phenotypes and their differential effects on core phenotypes. To gain further insight, we analyzed the phenotypic data. Overall, ASD males performed better than females in behaviors, cognition, language, social, and other domain (Supplementary Table S3). Linear regression analysis indicated that PRS is positively associated with social and expressive ability, while it was negatively associated with attention problems (Supplementary Table S5).

We also examined the distribution of the phenotype in unaffected siblings. In general, female siblings performed better than males in most assessments, which contrasts with the pattern observed in ASD probands. Female siblings had higher scores in communication, composite standard, daily living skills, and learning abilities, but also higher scores in repetitive behaviors and anxiety (Supplementary Table S4). We found a negative correlation between siblings’ PRS and composite standard score (beta = −3.65, *p* = 0.0009), daily living skills standard score (beta = −4.10, *p* = 0.0003), socialization standard score (−3.72, *p* = 0.0009). Higher PRS was associated with an aggravation of aggressive behavior, attention problems, and pervasive developmental problems (Supplementary Table S6).

### Identifying DNV contributions to ASD

**Figure 3:**
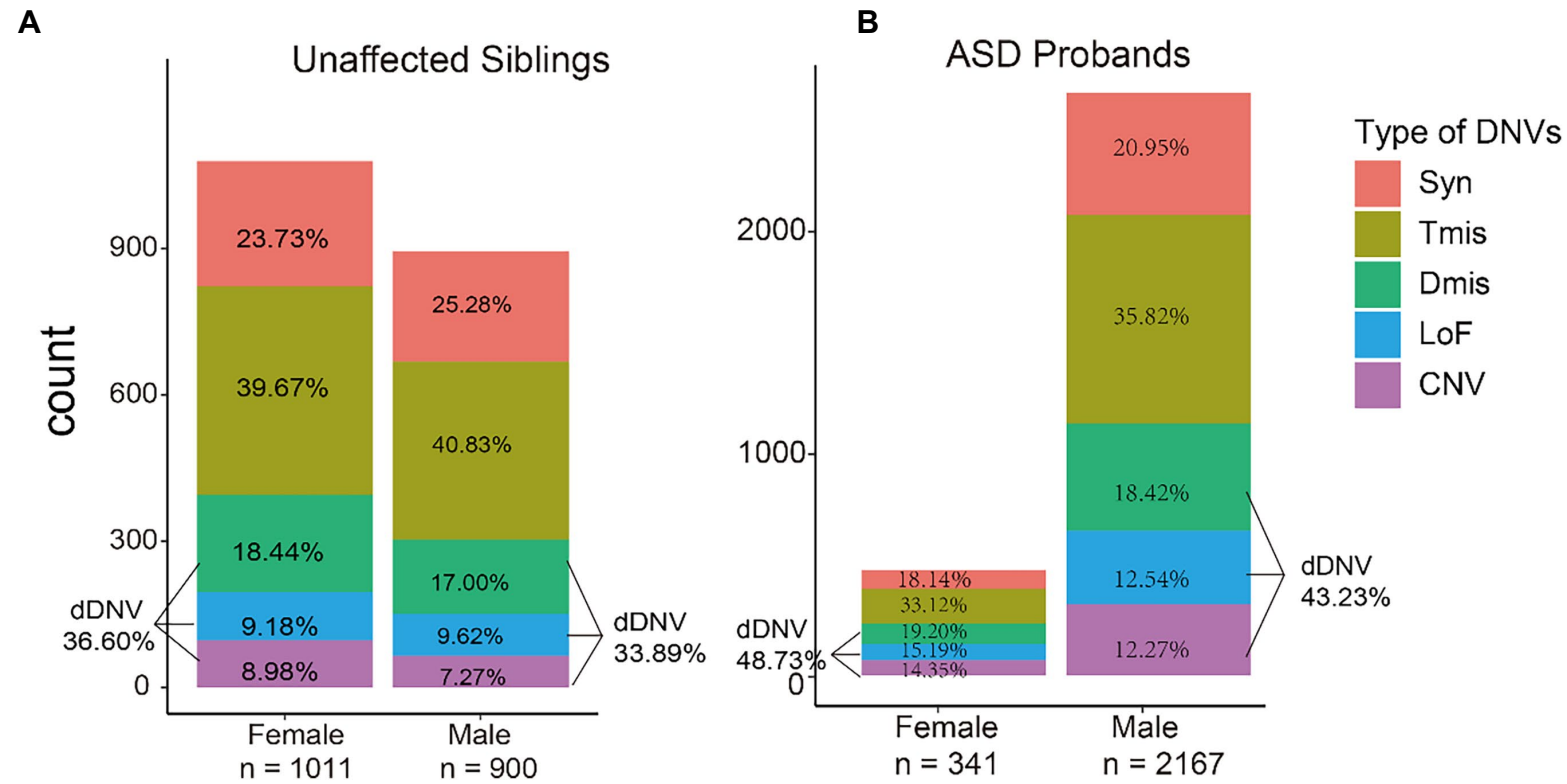
Proportion of *de novo* variant types in unaffected siblings and autism spectrum disorders (ASD) probands among males and females. The y-axis represents the number of variants in each group. Syn, synonymous variants; Tmis, tolerant missense variants; Dmis, deleterious missense variants; LoF, loss-of-function variants; CNV, copy number variants; dDNV, damaging DNV.

As expected, *de novo* damaging missense, loss-of-function variants, and copy number variants all showed an increased burden of ASD compared to synonymous variants. Intolerant missense variants used as negative controls did not affect ASD burden (*p* = 0.45). Over-enrichment of damaging DNVs was seen in ASD probands compared with unaffected siblings (odds ratio [OR] = 1.47, 95%CI = 1.26–1.71, *p* = 4.16E-7) (Supplementary Table S1). Both unaffected siblings and ASD probands showed a higher proportion of females carrying types of DNVs compared to males (2.71 and 5.50% in unaffected siblings and ASD probands, respectively) (Figure 3).

Further analysis was performed to investigate the relationship between DNVs and ASD core phenotypes. In contrast to PRS, all measurements in adaptive/cognitive behaviors exhibited negative correlations. DNVs are usually novel and more harmful than inherited variants because they are not subjected to strong natural selection. The relationships between other ASD core phenotypes and DNVs were also unclear(Supplementary Table S7).

We further analyzed the relationships between individuals carrying damaging DNVs and the broad autism phenotype in both ASD probands and their siblings. In all significant results, individuals carrying damaging DNVs had more severe symptoms than those without such variants (Supplementary Tables S8, S9).

### Interaction between polygenic risk and DNVs

**Figure 4:**
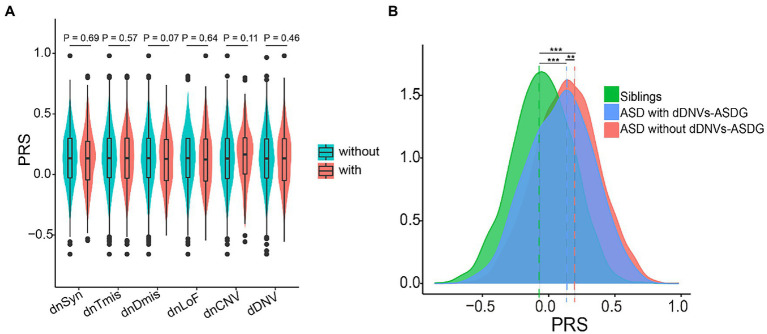
Interaction between polygenic risk and *de novo* variants (DNV). **(A)** Distribution difference of polygenic risk score (PRS) between probands with and without types of DNV. **(B)** Distribution difference of PRS between probands with and without dDNVs-ASDG. All polygenic scores were z-score scaled, and pairwise comparisons were performed *via* t-tests. **p < 0.01, ***p < 0.001. dnSyn, *de novo* synonymous variants; dnTmis, *de novo* tolerant missense variants (REVE <0.7); dnDmis, *de novo* deleterious missense variants (REVE ≥0.7); dnLoF, *de novo* loss-of-function variants; CNV, copy number variants; dDNV, damaging DNV; dDNVs-ASDG, dDNVs in ASD risk genes.

Polygenic risk and DNVs may interact additively to confer liability in ASD (4, 40). Therefore, we combined polygenic risk and DNVs to explore their interaction. We observed no significant associations between polygenic risk and any type of DNVs (Figure 4A). ASD is highly heterogeneous and has been linked to numerous susceptibility genes (41). We mapped damaging DNVs in 227 ASD risk genes (Supplementary Table S1). ASD probands not carrying damaging DNVs in ASD risk genes (*n* = 2059) had higher PRS than ASD probands carrying them (*n* = 190, *p* = 0.0098) (Figure 4B). In terms of ASD core and the broad phenotypes, probands with damaging DNVs in ASD risk genes showed lower scores than those classified as having a high DNV risk (Table 1; Supplementary Table S10).

**Table 1:** Descriptive statistics of ASD core phenotype measures for ASD probands without and with dDNVs-ASDG.

Core descriptive	Measures	Without dDNVs-ASDG mean (sd)	With dDNVs-ASDG mean (sd)	*p*
Adaptive/cognitive	Adaptive behavior composite	73.71 (12.05)	68.66 (11.31)	1.60E-08
	Verbal IQ	79.80 (30.97)	69.37 (29.63)	6.20E-06
	Nonverbal IQ	86.30 (25.73)	70.48 (22.97)	2.20E-16
	Full-scale IQ	83.06 (27.65)	68.42 (24.72)	3.20E-13
	Verbal mental age	84.81 (52.85)	74.34 (45.83)	3.30E-03
	Nonverbal mental age	92.85 (49.61)	75.53 (37.15)	8.60E-09
Language/social/communication	adi_r_soc_a_total	20.28 (5.7)	21.09 (5.49)	*0.052*
	ados_communication_social	13.26 (4.14)	13.43 (4.26)	0.62
	adi_r_comm_b_non_verbal_total	9.2 (3.47)	9.61 (3.29)	0.1
	adi_r_b_comm_verbal_total	16.45 (4.32)	16.92 (4.21)	0.17
Repetitive behaviors	adi_r_rrb_c_total	6.56 (2.51)	6.58 (2.46)	0.91
	rbs_r_overall_score	27.10 (17.45)	26.45 (15.8)	0.59
	ados_restricted_repetitive	3.91 (2.06)	4.15 (2.23)	0.17
Behavioral problems	abc_total_score	45.86 (25.52)	48.03 (25.56)	0.27
	cbcl_2_5_externalizing_t_score	57.83 (10.68)	58.85 (12.56)	0.61
	cbcl_2_5_internalizing_t_score	61.24 (8.92)	60.83 (8.91)	0.78
	cbcl_6_18_externalizing_t_score	56.07 (10.52)	57.96 (10.38)	0.12
	cbcl_6_18_internalizing_t_score	60.25 (9.76)	59.39 (9.04)	0.28

*p* values below 0.05 were highlighted in italics; ASD, autism spectrum disorder; dDNVs-ASDG, damaging de novo variants in ASD risk genes; ADI-R, Autism Diagnostic Interview-Revised (29); ADOS, Autism Diagnostic Observation Schedule (42); ABC, Aberrant Behavior Checklist (43).

### Multiple factors combined to explain ASD adaptive and cognitive behaviors

**Figure 5:**
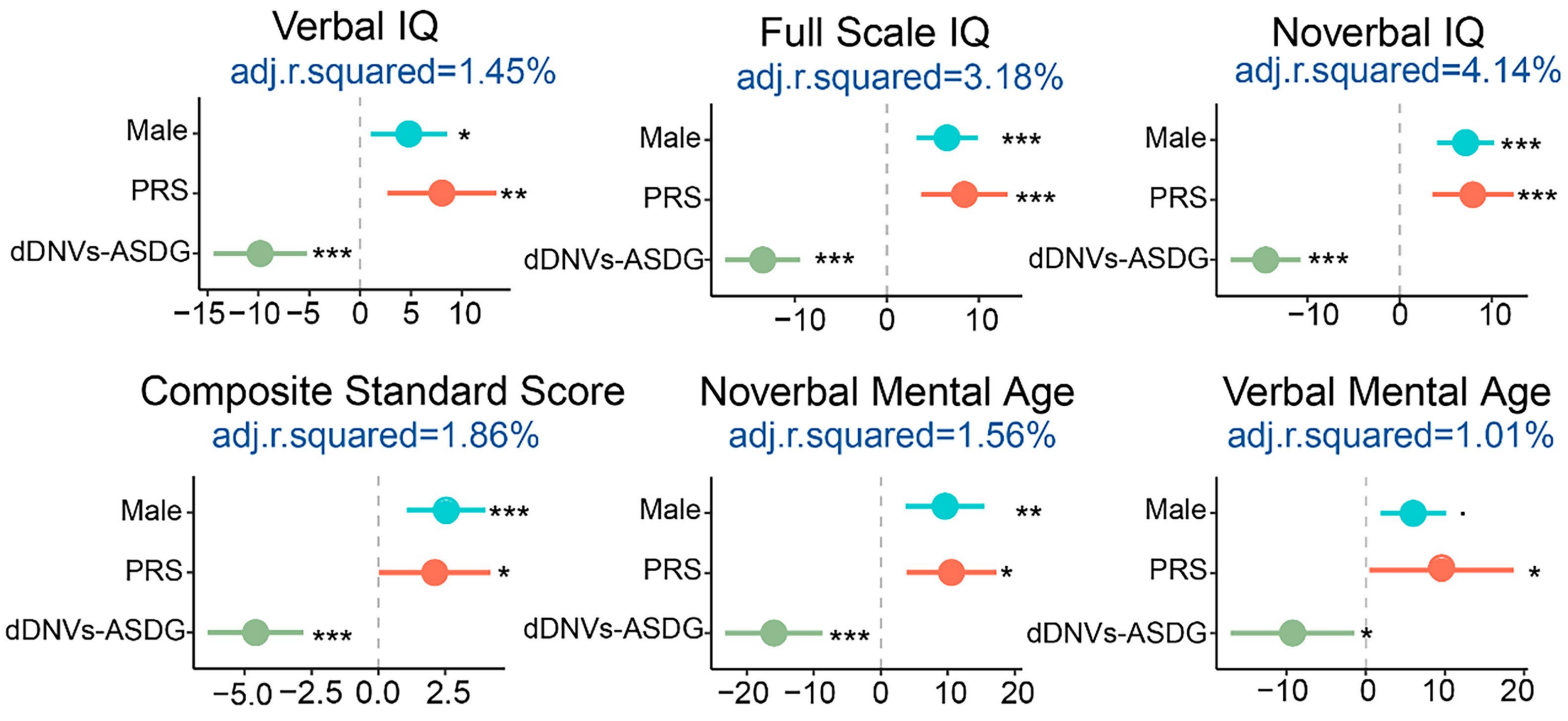
Estimation of the impact sizes and liability of sex, polygenic risk scores (PRS), and damaging *de novo* variants in ASD risk genes (dDNVs-ASDG) on adaptive/cognitive behaviors. The x-axis shows the Pearson correlation coefficient and the adjusted *R*^2^ represents the liability scale of the phenotypes explained by the risk factors. IQ, intelligence quotient. *p < 0.05, **p < 0.005, ***p < 0.001.

According to the above results, adaptive/cognitive behaviors were affected by sex, polygenic risk, and damaging DNVs of ASD risk gene (*p* < 0.05), with lower scores indicating more severe adaptive/cognitive phenotypes. The female had lower adaptive/cognitive behavior scores than males. PRS was positively associated with these scores while damaging DNVs (including those in ASD risk genes) were negatively associated with them. When PRS, damaging DNVs in ASD risk genes, and sex was combined, they were able to explain a small portion of the variance in adaptive/cognitive phenotypes, specifically 4% of nonverbal IQ, 3% of full-scale IQ, 1.6% of verbal IQ, and 1.9% of adaptive behavior composite. It is important to note that there are likely other factors at play, including undetected genetic factors and environmental risks that also need to be considered (Figure 5).

## Discussion

In this study, we investigated the effects of common polygenic risk and DNVs on ASD individually, and in combination. Previous studies have shown that both PRS and damaging DNVs in probands had a greater overall genetic burden compared to unaffected siblings (1, 15). The genetic susceptibility of any disease may be caused by many common small-effect genetic variants, rare variants of large effect, or a combination of the two (44). Then, we explored the interaction between PRS and DNVs. Although there were no significantly different PRS among probands with or without damaging DNVs, the PRS of individuals carrying damaging DNVs in ASD risk genes was lower than that of non-carriers, which implied that damaging DNVs in ASD risk genes have a greater genetic load of ASD risk. A recent study also observed similar results that ASD cases carrying damaging DNVs reduced PRS, in the presence of damaging DNV, the less polygenic risk is required to meet diagnostic criteria for ASD (4).

Genetic risks range from rare to common and affect genes in a combined or individual manner that can result in ASD core and broad phenotypes (16, 22). Our study found that the effects of polygenic risk and damaging DNVs on ASD phenotypes were inconsistent. In the general population, PRS for ASD is associated with many positive traits (45). We noted ASD subjects with higher PRS tended to show improvements in adaptive/cognitive behaviors and communication/expressive/learning ability but also aggravated attention problems. While ASD subjects carrying damaging DNVs tended to have more severe ASD phenotypes. Most of the significant results were observed in adaptive/cognition behaviors. Previous studies have shown that genetic influences on language, social communication, restricted/repetitive behaviors, and the symptoms can decrease with age (46, 47). We speculated that environmental factors play an important role in social communication and restricted/repetitive behaviors, resulting in a reduced effect of genetic risk on these ASD core phenotypes. We also investigated the relationship between PRS, damaging DNVs, and the broad autism phenotypes in siblings. Our findings suggest that siblings with higher PRS for ASD and damaging DNVs tend to have more severe phenotypes, with lower social scores, increased aggressive behavior, and attention problems.

Male sex is a strong risk factor for ASD. Our study showed that female ASD proband carried a greater burden of both common polygenic risk and damaging DNVs, supporting the female protective effect on ASD. Females may have a higher tolerance for ASD risk without receiving an ASD diagnosis (3). Meanwhile, we also observed that females with ASD tend to have more severe symptoms than males with ASD, which may suggest that females require more severe symptoms to be diagnosed with ASD. A recent epidemiological survey of ASD indicated that the male/female ratio decreased in the last decade, possibly due to increasing clinical attention to ASD in females (5). However, diagnostic biases could not adequately explain the male-biased sex ratio, the ratio that has been remarkably stable over time (48). Another explanation is that males with only one X chromosome are more likely to express ASD if they inherit a mutation in an X-linked gene associated with the disorder. Although rare genetic events causing ASD have been identified on the X chromosome, such as mutations in the NLGN3, NLGN4X, ARX, MECP2, and FMR1 genes, current evidence does not support the sex chromosome risk model which proposes that common risk factors of strong effect for ASDs lie on the X or Y chromosomes (49, 50). Sex hormones like testosterone and estradiol may contribute to male bias in ASD and predict future ASD-related behaviors, but their impacts require further study (51). In summary, the mechanisms underlying why females are more tolerant of ASD’s genetic risk remain unclear. We need a new conceptual framework to understand the complex factors that contribute to ASD risk, and large-scale studies analyzing brain development and function at a molecular level can help uncover the mechanisms behind male–female differences in ASD (3, 52).

Our research suggests that PRS, damaging DNVs in ASD risk genes, and sex only explain 1–4% of the liability of adaptive/cognitive behavior, demonstrating that ASD is a highly complex and heterogeneous condition. It is important to consider that ASD core phenotypes may involve both genetic and non-genetic factors that contribute to the phenotypic spectrum. Epigenetic dysregulation in sensitive developmental periods and brain special regions can also affect behavioral phenotypes (53). Additionally, environmental exposures, such as maternal/paternal age, metabolic syndrome features, and the use of antidepressants, can influence ASD development (54, 55). Their combined effect may have greater consequences. Our study highlights that integrating multiple risk factors can improve our understanding of the high genetic and phenotypic heterogeneity of ASD.

## Data availability statement

The original contributions presented in the study are included in the article/Supplementary material, further inquiries can be directed to the corresponding author/s.

## Ethics statement

The studies involving human participants were reviewed and approved by Sfari Base. Written informed consent to participate in this study was provided by the participants' legal guardian/next of kin.

## Author contributions

LD planned, analyzed the data, created the figures, and drafted the manuscript. JL and KX designed and conceived this study and supervised the work. LD, LX, JL, and KX wrote and revised the manuscript. LD, YW, XW, TL, QZ, GZ, BL, LX, KX, and JL participated sufficiently in drafting the manuscript or revising the crucial intellectual content. All authors contributed to the article and approved the submitted version.

## Funding

This study was funded by the Hunan Youth Science and Technology Innovation Talent Project (grant number: 2020RC3060 to JL, 81730036, 82101246 to KX and LX) and the Natural Science Foundation of Hunan Province (grant number: 2021SK1010, 2021JJ40813 to KX and LX).

## Conflict of interest

The authors declare that the research was conducted in the absence of any commercial or financial relationships that could be construed as a potential conflict of interest.

## Publisher’s note

All claims expressed in this article are solely those of the authors and do not necessarily represent those of their affiliated organizations, or those of the publisher, the editors and the reviewers. Any product that may be evaluated in this article, or claim that may be made by its manufacturer, is not guaranteed or endorsed by the publisher.

## Footnotes

^1^ http://brant.brainnetome.org/en/latest/
