# Supplementary material for: Interactions of genetic risks for autism and the broad autism phenotypes
Source: Front Psychiatry. 2023 Mar 21;14:1110080. doi: 10.3389/fpsyt.2023.1110080 (PMC10123509; doi:10.3389/fpsyt.2023.1110080)

**Figure S1.** Quantile-Quantile Plot of P-values distribution from the transmission disequilibrium test.


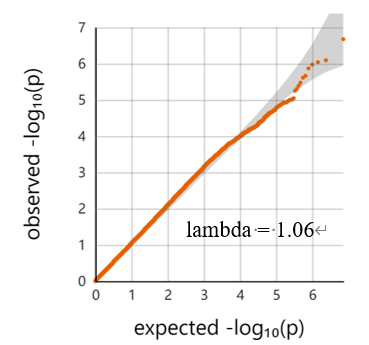


**Figure S2.** Distribution of polygenic risk score (PRS) between the sex of ASD probands in their siblings.


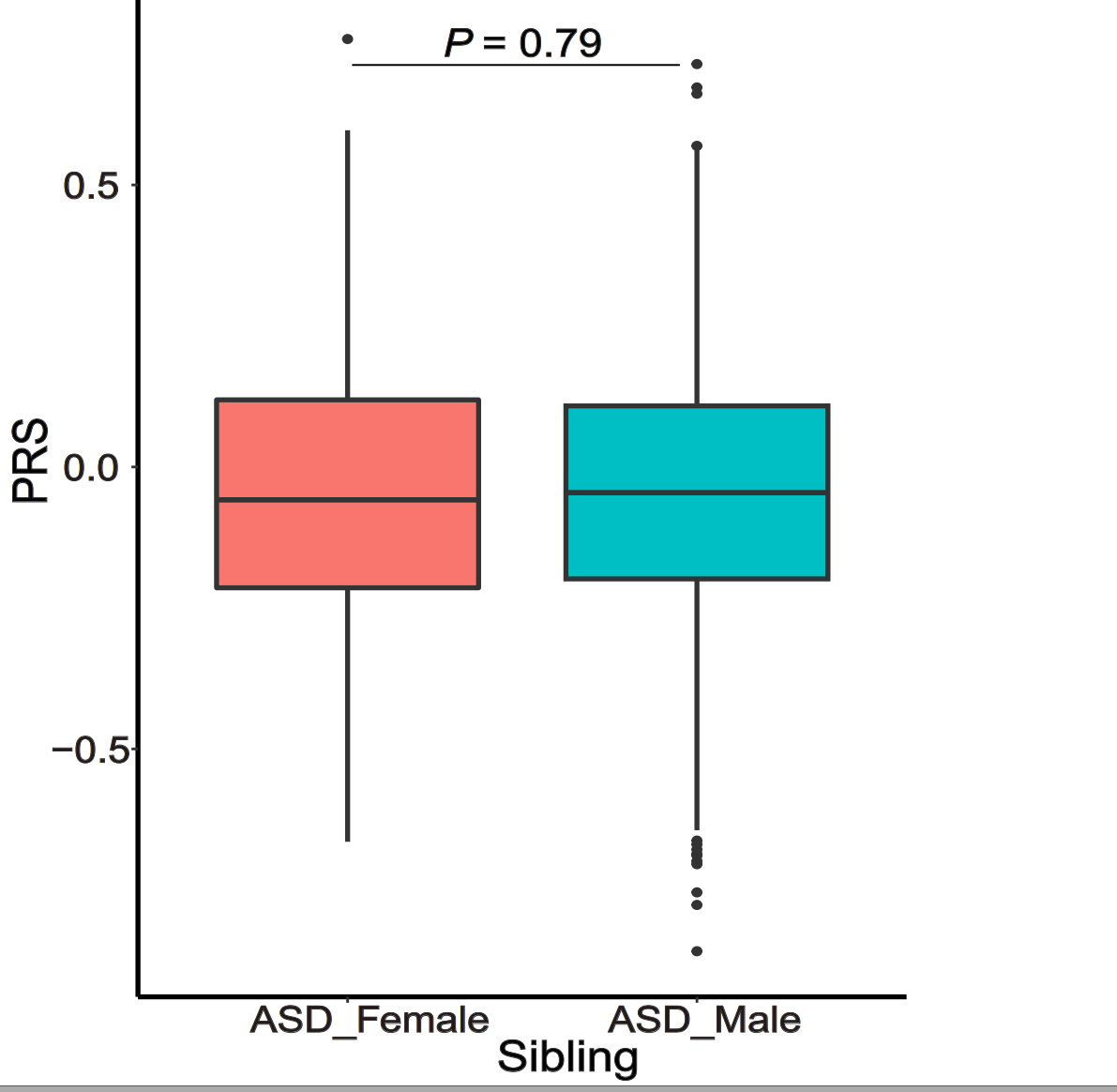


**Figure S3.** The bias of polygenic risk score (PRS) and cognitive impairment between males and females in ASD probands.

**
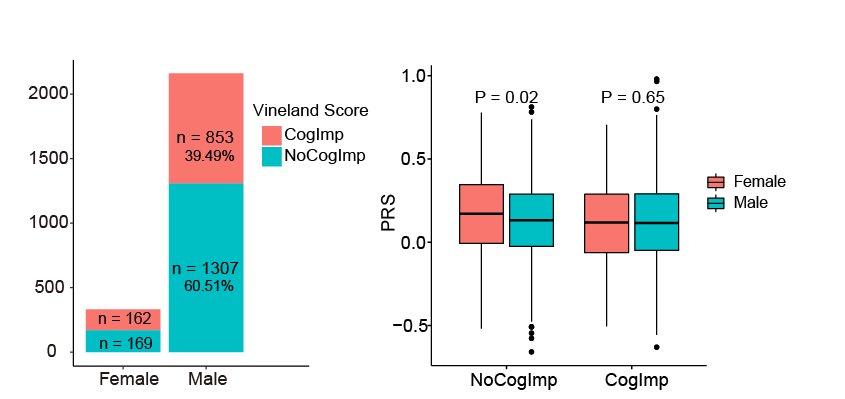
**

**Figure S4**. The relationship between adaptive/cognitive behaviors and polygenic risk score (PRS) in ASD probands.


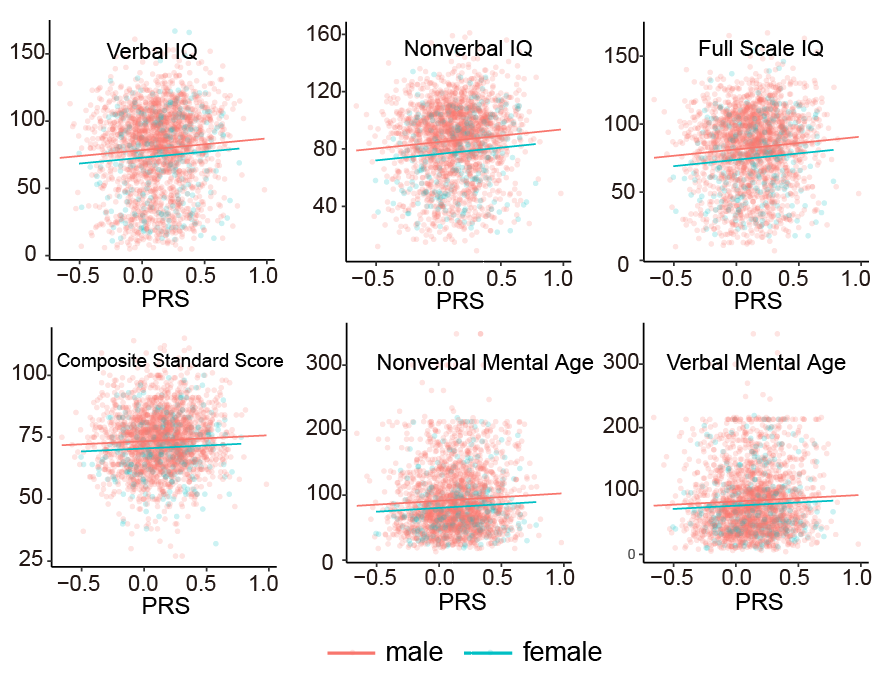

Supplement: Supplementary file 2 [file Data_Sheet_1.docx]
